# Diets Differently Regulate Pulmonary Pathogenesis and Immune Signaling in Mice during Acute and Chronic *Mycobacterium tuberculosis* Infection

**DOI:** 10.3390/life13010228

**Published:** 2023-01-13

**Authors:** Neelam Oswal, Hariprasad Thangavel, Kezia Lizardo, Dhanya Dhanyalayam, Tabinda Sidrat, Padmini Salgame, Jyothi F. Nagajyothi

**Affiliations:** 1 Center for Discovery and Innovation, Hackensack Meridian Health, Nutley, NJ 07110, USA; 2 Department of Medicine, Center for Emerging Pathogens, Rutgers-New Jersey Medical School, Newark, NJ 07103, USA

**Keywords:** *Mycobacterium tuberculosis*, C57BL/6 mice, diet, adiponectin, adipogenesis, lipolysis, necrosis, immune cell, macrophage, inflammation

## Abstract

Tuberculosis (TB) caused by *Mycobacterium tuberculosis* (Mtb) infection persists as a leading cause of mortality and morbidity globally, especially in developing and underdeveloped countries. The prevalence of TB-DM (diabetes mellitus) is higher in low- and middle-income countries where TB and DM are most prevalent. Epidemiological data suggest that slight obesity reduces the risk of TB, whereas DM increases the risk of pulmonary TB. Diets can alter the levels of body fat mass and body mass index by regulating systemic adiposity. Earlier, using a transgenic Mtb-infected murine model, we demonstrated that loss of body fat increased the risk of pulmonary bacterial load and pathology. In the present study, we investigated whether increased adiposity alters pulmonary pathology and bacterial load using C57BL/6 mice infected with HN878 Mtb strain and fed a medium-fat diet (MFD). We analyzed the effects of MFD on the lung during acute and chronic infections by comparing the results to those obtained with infected mice fed a regular diet (RD). Histological and biochemical analyses demonstrated that MFD reduces bacterial burden by increasing the activation of immune cells in the lungs during acute infection and reduces necrosis in the lungs during chronic infection by decreasing lipid accumulation. Our data suggest that slight adiposity likely protects the host during active TB infection by regulating immune and metabolic conditions in the lungs.

## 1. Introduction

*Mycobacterium tuberculosis* (Mtb) causes tuberculosis (TB), which infects around a quarter of the world’s population and is a leading cause of death globally. Despite the ongoing efforts to control TB, there are new challenges, such as multi-drug resistant TB, lack of effective vaccines, and HIV-TB co-infection, which pose roadblocks to controlling this disease. There is also an association between type 2 diabetes mellitus and TB [1,2,3]. Previously, we reported that Mtb infection increases insulin resistance and stress-induced hyperglycemia [4]. Recent studies suggest that diabetes can increase the risk of contracting TB by three-fold and impact TB treatment [5]. The combination of diabetes and TB may cause severe inflammatory and metabolic dysfunctions. Therefore, a clear understanding of immune and metabolic responses is required to design better strategies to control TB [6].

Mtb primarily resides in the lungs but can also be present in extrapulmonary tissues, such as fat tissue [7]. Previous studies from our laboratory have shown that Mtb can infect adipocytes and alter the immune and metabolic environment in adipose tissue [8]. Adipocytes play a major role in regulating whole body immune and metabolic homeostasis [9]. Using Mtb-infected transgenic FAT-ATTAC (fat apoptosis through targeted activation of caspase 8) mice, we demonstrated that fat ablation during Mtb H37Rv acute infection increases pulmonary bacterial loads as compared to fat-unablated mice [10]. Our previous report suggested that a loss of body fat may increase the risk of pulmonary TB pathology and reactivation of TB [10]. Clinical studies suggest that body mass index (BMI) positively correlates with a reduced risk of pulmonary TB and an increased rate of treatment success [11]. In the current study, we examined whether a slight increase in adiposity reduces pulmonary pathology during acute and chronic Mtb infections using Mtb HN878 aerosol infected C57BL/6 mice fed either a medium-fat diet (MFD; 50% carbohydrate calories, 20% calories of protein, and 30% calories of fat) or a control regular diet (RD; 70% carbohydrate calories, 20% calories of protein, and 10% calories of fat). We demonstrated that these diets differently alter pulmonary pathology and bacterial loads between acute and chronic Mtb infections by regulating infiltration of immune cells, inflammation, adipogenesis, accumulation of lipid droplets, and lipolysis in the lungs. We also examined whether uninfected and Mtb-infected adipocytes differently regulate adipogenic and inflammatory signaling in uninfected and Mtb-infected macrophages. Our data suggest that adiponectin, an anti-inflammatory adipogenic protein highly expressed and secreted by adipocytes, regulates macrophage inflammatory signaling, which is an important mechanism that determines the polarization and activation of macrophages during Mtb infection.

## 2. Materials and Methods

### 2.1. Bioethics and Biosafety

All animal experimental protocols were approved by the Institutional Animal Care and Use Committee (IACUC) and Institutional Biosafety Committees of Center for Discovery and Innovation (CDI)-Hackensack University Medical Center and adhered to National Research Council guidelines.

### 2.2. Animal Model and Experimental Design

The C57BL/6 (4 weeks old, male, *n* = 40; female, *n* = 14) mice were purchased from the Jackson Laboratory and housed in the BSL2 facility at CDI-Hackensack University Medical Center. Mice were maintained on a 12 h light/12 h dark cycle and housed in groups of three to four mice per cage. Mice were divided into two groups at 4 weeks of age (male, *n* = 20 and female, *n* = 14 per group) and fed on either a medium-fat diet (MFD; 30% fat calories D20072301 Research Diets, Inc., New Brunswick, NJ, USA) or a low-fat control diet (RD; 10% calories of fat). Half of the RD male (*n* = 10) and MFD male mice (*n* = 10) and all female mice (RD, *n* = 7; MFD, *n* = 7) were aerosol infected with M. tuberculosis HN878 in BSL3 facility at Center for Discovery and Innovation of Hackensack University Medical Center, 4 weeks after putting on diet. Briefly, Mtb aerosols were generated by a Glas-Col Inhalation Exposure System (Glas-Col) with a 5 mL bacterial suspension of about 3 × 10^6^ bacilli/mL in PBS containing 0.04% Tween 80, and the mice were exposed to the aerosol for 30 min, which results in seeding of approximately 100 colony forming unit (CFU) per lung. Uninfected male mice were fed on either MFD (*n* = 10) or RD (*n* = 10) and used as respective controls in the experiment. Mice were sacrificed at either 30 days post-infection (DPI) (acute model) or 90 DPI (chronic model) and lungs and WAT (epididymal fat pads) were harvested. Portions of the lung tissues were homogenized in phosphate-buffered saline-Tween (PBST), and serial dilutions of the homogenates were plated onto Middlebrook 7H10 agar (Difco BD, Sparks, MD, USA) to determine the number of bacterial CFU. Portions of the harvested lung tissue and white adipose tissue (WAT) were fixed in 10% formalin for histological analysis. Portions of tissues were also stored immediately at −80 °C for protein extraction. A flowchart describing the experimental design is presented (Supplementary Figure S1a).

### 2.3. Histological Analysis

Freshly harvested tissues were fixed with phosphate-buffered formalin for a minimum of 48 h and then embedded in paraffin wax for histological analysis. The slides were stained with hematoxylin and eosin stain (H&E), and the images were captured as previously published [12]. Four to six images per section of each lung were scored blindly. For each sample, histologic evidence of pathology was classified in terms of the presence of infiltrated immune cells, lipid droplets, and foamy macrophages and was graded on a 7-point scale ranging from 0 to 6. Auramine-rhodamine (AR) staining of the lung and adipose tissue sections was performed, and the images were captured [4]. Four to six images per section of each lung were quantitated for fluorescence intensity by Image J. Immunohistochemical analysis (IHC) was performed on the formalin-fixed lung using CD4 specific rabbit polyclonal antibody (#NBP1-19371, Novus Biologicals), CD8 specific rabbit polyclonal antibody (#NBP2-29475, Novus Biologicals), F4/80-specific rabbit polyclonal (#ab100790, Abcam), and IFNγ-specific rabbit polyclonal antibody (#BS-0480R, Bioss Antibodies) as described earlier [13]. We analyzed the antibody-stained slides with semi-quantitative scoring system and graded them on a 7-point scale ranging from 0 to 6.

### 2.4. Immunoblot Analysis

Protein lysates of the lung and WAT samples were prepared and immunoblot analysis was performed as described earlier [10]. Briefly, tissue lysates of the lungs and WAT were prepared by homogenizing the tissue using a handheld homogenizer after the addition of cell lysis buffer (#9803, Cell Signaling Technology) containing Pierce protease inhibitor cocktail (#A32963, ThermoFisher Scientific). The homogenate was then incubated on ice for 10 min before clarification by centrifugation for 15 min at 14,000× *g* in a cold microfuge. The supernatant was recovered, and the protein concentration was quantified using Pierce BCA protein assay kit (#23225, ThermoFisher Scientific). An amount of 30 µg total protein from each sample was loaded and resolved on SDS-PAGE and then transferred onto nitrocellulose membrane for immunoblot analysis. The blots were probed with the following primary antibodies: PPARα-specific mouse monoclonal antibody (#MA1-822, ThermoFisher Scientific), Perilipin 1-specific rabbit monoclonal antibody (#9349, Cell Signaling Technology), Phospho-Perilipin 1 (Ser522)-specific mouse monoclonal antibody (#4856, Vala Sciences), F4/80-specific rat monoclonal (#NB600-404, Novus Biologicals), adiponectin-specific mouse monoclonal antibody (#ab22554, Abcam), IFNγ-specific rabbit monoclonal antibody (#ab133566, Abcam), TNFα-specific rabbit polyclonal antibody (#ab6671, Abcam), CD4 specific rabbit polyclonal antibody (#NB100-56457SS, Novus Biologicals), CD8 specific rabbit polyclonal antibody (#NBP2-29475, Novus Biologicals), BNIP3 specific rabbit polyclonal antibody (#44060, Cell signaling Technology), Caspase-3-specific rabbit polyclonal antibody (#9662, Cell signaling Technology), IL-6-specific mouse monoclonal antibody (#66146-1-Ig, Proteintech), and IL-10-specific mouse monoclonal antibody (#60269-1-Ig, Proteintech). Horseradish peroxidase (HRP)-conjugated anti-mouse immunoglobulin (#7076, Cell Signaling Technology), HRP-conjugated anti-rabbit immunoglobulin (#7074, Cell Signaling Technology), and HRP-conjugated anti-rat immunoglobulin (#112-035-003, Jackson ImmunoResearch) were used as appropriate secondary antibodies to detect chemiluminescent signal using Invitrogen iBright Imaging Systems. Guanosine nucleotide dissociation inhibitor (GDI)-specific rabbit polyclonal antibody (#71–0300, Invitrogen, CA, USA) was used to normalize protein loading.

### 2.5. Analysis of Circulatory Cytokines and Serum Lipids

Serum levels of triglyceride (#10010303, Cayman Chemical) and total cholesterol, HDL, and LDL/VLDL (#ab65390, Abcam) were analyzed using colorimetric kits according to the manufacturer’s protocol. Serum levels of cytokines such as IL-6 (#431307, BioLegend), IFNγ (#KMC4021, Invitrogen), and TNFα (#BMS607-3, Invitrogen) were measured in male acute and chronic infected mice using ELISA kits following the manufacturer’s protocol. 

### 2.6. In Vitro Trans-Well Co-Culture Experiment

To evaluate the effect of adipocytes on the inflammatory status of macrophages during Mtb infection, an in vitro experiment was set up with RAW 264.7 macrophages and 3T3-L1 adipocytes co-cultured in a trans-well culture system. The first group was uninfected macrophages cultured in a 12-well plate (control). In the second group, macrophages were infected with 1:5 multiplicity of infection (MOI) with HN878 Mtb strain. For the rest of the groups, 3T3-L1 pre-adipocytes were seeded in the trans-well of a 12-well plate and differentiated into adipocytes until day 14 and the macrophages were seeded in 12-well plates. Then, both cells were put together in a co-culture system. In the third group, the macrophage cells were infected with 1:5 MOI infected with HN878 strain and adipocytes were in the trans-well. The fourth group consisted of infected macrophages and adipocytes in the trans-well treated with 10 ng/mL recombinant TNF to induce cell death in the adipocytes. In the fifth group, uninfected adipocytes in the trans-well and macrophages in the 12-well plate were placed in a co-culture system. The sixth group comprised of adipocytes infected with 1:5 MOI with HN878 strain in the trans-well and macrophages in the bottom compartment. The cells were co-cultured for a period of 24 h (acute) after infection, following which the cells were collected in lysis buffer for protein analysis. A layout of the trans-well co-culture experiment is presented (Supplementary Figure S1b).

### 2.7. Statistical Analysis

Statistical analyses were performed using GraphPad Prism version 9.4.1 (GraphPad Software, Inc., La Jolla, CA, USA). Comparisons between groups were made using unpaired Student’s *t*-test. The error bars represent standard error of the mean. Values of * *p* < 0.05, ** *p* < 0.01, and *** *p* < 0.001 between indicated groups were considered statistically significant.

## 3. Results

We previously used H37Rv aerosol-infected transgenic FAT-ATTAC mice to demonstrate that loss of body fat increases pulmonary bacterial load and pathology due to impaired activation of immune cells [10]. In the current study, we analyzed whether MFD-induced adiposity alters pulmonary pathology and Mtb burden in the lungs during acute (30 DPI) and chronic (90 DPI) infections in HN878-infected mice. We measured the body weights of the acute and chronic groups of mice at 0 and 30 DPI and at 0 and 90 DPI, respectively. The weights of infected RD-fed male mice significantly decreased (*p* < 0.05) compared to infected MFD-fed male mice during acute infection. The weights of MFD-fed infected female mice were significantly decreased compared to MFD-fed uninfected female mice. During chronic infection, we observed significant differences in body weight between males and females and between mice fed with MFD and RD. In males, the weights of infected mice were significantly decreased (*p* < 0.01) as compared to diet-matched control mice, whereas in females, there was no significant difference between the weights of control and infected diet-matched groups. As expected, both in males and females, mice fed with MFD showed increased body weight as compared to mice fed with RD (Supplementary Figure S1c).

### 3.1. MFD Alters Lung Histopathology during Acute and Chronic Mtb Infection in a Murine TB Model

**Figure 1:**
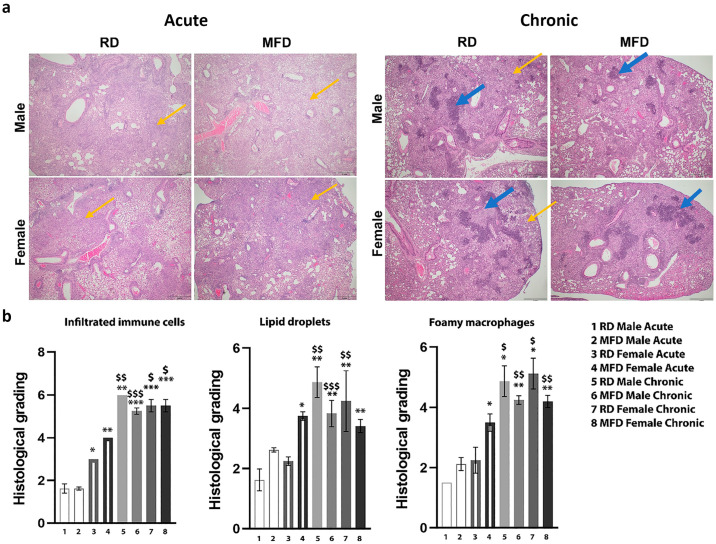
MFD alters lung histopathology during acute and chronic Mtb infection in a murine TB model. (**a**) Histological analysis of the lungs in H&E sections of lungs in acute and chronic Mtb infection in male and female mice. The images are at 20× magnification, scale bar = 2 mm. Yellow arrows indicate infiltrated immune cells and blue arrows indicate mature granulomas. (**b**) Histological grading of lung pathology classified as infiltrated immune cells, lipid droplet accumulation, and foamy macrophages. Each class was graded on a 7-point scale ranging from 0 to 6 as discussed in the Materials and Methods section. The error bars represent the standard error of the mean. * *p* < 0.05, ** *p* < 0.01, and *** *p* < 0.001 with respect to RD male acute infected mice. $ *p* < 0.05, $$ *p* < 0.01, and $$$ *p* < 0.001 between similar groups in acute and chronic infection.

Histological analysis of H&E-stained lung sections demonstrated significant differences in pathology between the diets, sexes, and stages of infection (acute vs. chronic), including the levels of infiltration of immune cells, accumulation of lipid droplets, and presence of foamy macrophages. During acute infection, the levels of infiltrated immune cells were greater in female mice compared to male mice irrespective of the diet (Figure 1). However, the levels of infiltrated immune cells significantly increased in the lungs of chronic mice compared to acute mice irrespective of mouse diet or sex (Figure 1). We observed higher levels of infiltrated T cells and B cells and formation of matured and/or necrotic granulomas in the lungs of chronically infected mice. H&E sections showed significantly increased levels of organized granulomas, foamy macrophages, and accumulation of lipid droplets in the lungs during chronic infection compared to acute infection irrespective of mouse diet or sex (Figure 1 and Supplementary Figure S2). The levels of lipid droplets and foamy macrophages were higher in RD-fed mice compared to MFD-fed mice during chronic infection, as shown by histological images (Figure 1 and Supplementary Figure S2).

### 3.2. MFD Alters Bacterial Load in the Lungs of Mtb-Infected C57BL/6 Mice during Acute Infection

**Figure 2:**
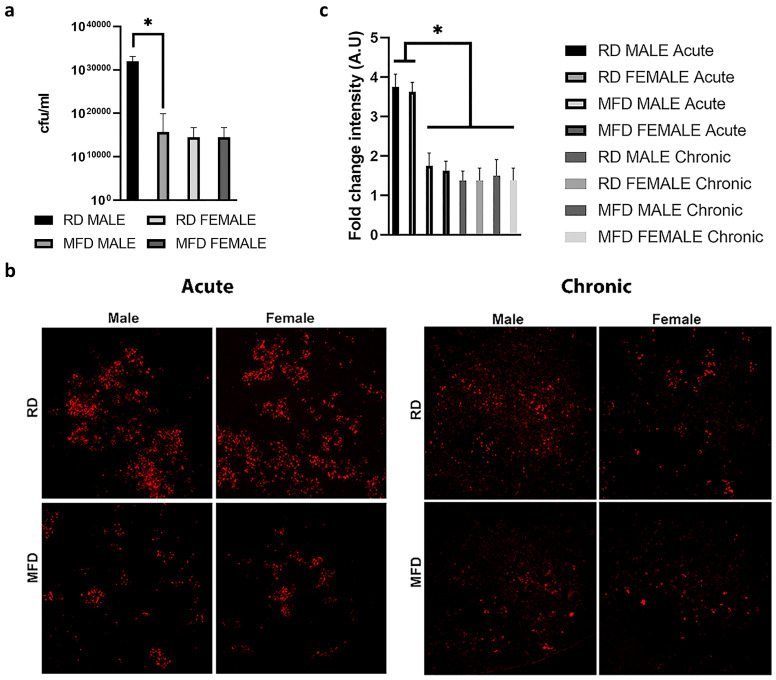
MFD alters bacterial load in the lungs of HN878 infected C57BL/6 mice during acute infection. (**a**) CFU in lung homogenates of acutely infected mice. (**b**) AR staining of lung sections in acutely and chronically infected mice. The images are at 20× magnification. (**c**) Fold change in intensity in AR staining in acutely and chronically infected mice. The error bars represent standard error of the mean. * *p* < 0.05 between indicated groups.

We analyzed the effect of MFD on lung bacterial burden in aerosol Mtb-infected (HN878 strain, 10^6^ bacilli) acutely infected mice by assessing the CFU in lung homogenates at 30 DPI as previously described [8]. The average CFU count was significantly higher in RD-fed mice compared to MFD-fed mice at 30 DPI; however, we observed no significant difference between the groups at 90 DPI (Figure 2a). Auramine-rhodamine staining of lung sections indicated a significantly increased (*p* < 0.01) bacterial load in the lungs of RD-fed mice compared to MFD-fed mice at 30 DPI (Figure 2b). The bacteria were mostly spread throughout the lung sections in RD-fed infected mice but were mostly clustered in small groups in MFD-fed infected mice at 30 DPI (Figure 2b). The levels of bacterial load significantly decreased in the lungs of chronically infected mice (90 DPI) compared to acutely infected mice (30 DPI). However, no significant difference in bacterial load between RD- and MFD-fed mice was detected at 90 DPI. Additionally, no significant differences in bacterial load in the lungs between male and female mice were detected (Figure 2c).

### 3.3. MFD Alters the Level of Infiltrated Immune Cells and Inflammatory Markers in the Lungs during Acute and Chronic Mtb Infection

**Figure 3:**
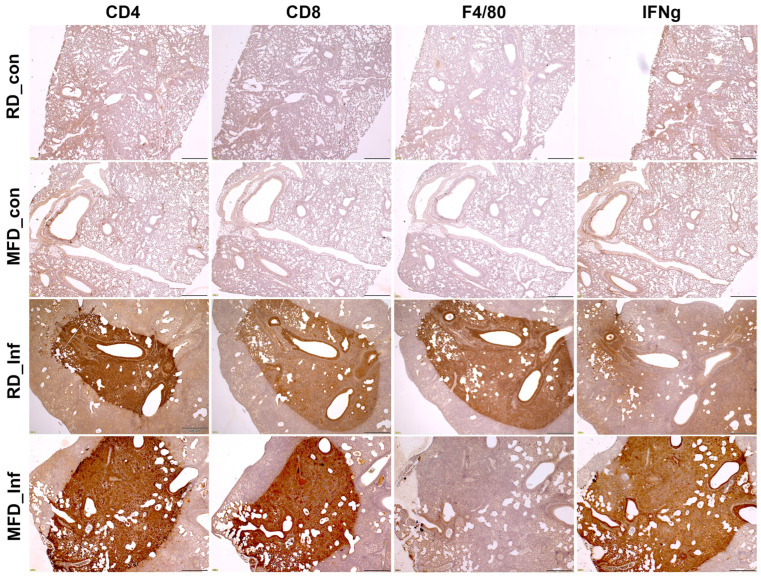
Representative immunohistochemistry (IHC) images of the lung sections of mice during acute Mtb infection (30 DPI) stained by CD4^+^, CD8^+^, F4/80^+^, and IFNγ antibodies. (RD_con—uninfected mice fed an RD; MFD_con—uninfected mice fed an MFD; RD_Inf—infected mice fed an RD; and MFD_Inf—infected mice fed an MFD). Magnification-4×; Scale bar–100 µ. IHC sections were graded and presented as a bar graph (see Supplementary Figure S3a).

We analyzed the levels of infiltrated immune cells, such as CD4^+^ cells, CD8^+^ cells, and F4/80^+^ cells, in the lungs by IHC and Western blotting analysis. The levels of CD4, CD8, and F4/80 were not significantly altered between diet-matched infected male and female mice (Supplementary Figure S3). Therefore, for further analysis, we used only male mice because males are more susceptible to TB compared to females [14]. The semi-quantitative IHC analyses demonstrated significantly increased levels of CD4^+^, CD8^+^, and F4/80^+^ cells and IFNγ levels in the lungs of infected mice during acute and chronic infection compared to their respective diet-matched uninfected mice (Figure 3 and Supplementary Figure S4a). RD-fed infected mice showed increased levels of F4/80^+^ cells in the lungs compared to MFD-fed mice during acute infection. MFD-fed infected mice showed increased CD4^+^ and CD8^+^ levels in the lungs compared to RD-fed infected mice during acute infection (Figure 3 and Supplementary Figure S4b). IHC analysis did not show significant differences in the levels of CD4^+^, CD8^+^, and F4/80^+^ in between the RD-fed infected and MFD-fed infected chronic mice (Supplementary Figure S4a). IHC analysis also demonstrated significantly increased IFNγ levels in the lungs of MFD-fed infected mice compared to RD-fed infected mice during both the acute and chronic stages of infection (Figure 3 and Supplementary Figure S4a).

To re-evaluate the IHC data, we also performed Western blotting analysis using the lung lysates and probed for CD4^+^, CD8^+^, and F4/80^+^ and various other cytokines. In male mice, during acute infection, the levels of CD4^+^ cells and CD8^+^ significantly increased in MFD-fed infected mice compared to RD-fed infected mice as demonstrated by Western blotting analysis (Supplementary Figure S4c). The levels of F4/80^+^ cells significantly increased in RD-fed infected mice compared to RD-fed uninfected mice during acute infection (Supplementary Figure S4c). The levels of F4/80 significantly decreased in MFD-fed infected mice compared to RD-fed infected mice during acute infection (Supplementary Figure S4c). We further analyzed the active status of infiltrated immune cells during infection by measuring the levels of pro-inflammatory markers such as IFNγ, TNFα, and IL-6 in the lungs by Western blotting. The levels of IFNγ and IL-6 significantly increased in the lungs of infected mice compared to uninfected mice both in RD- and MFD-fed groups during acute infection (Supplementary Figure S4c). The levels of IFNγ and IL-6 further significantly increased in MFD-fed infected mice compared to RD-fed infected mice during acute infection. The levels of TNFα increased in infected mice compared to uninfected mice during acute infection (Supplementary Figure S4c).

**Figure 4:**
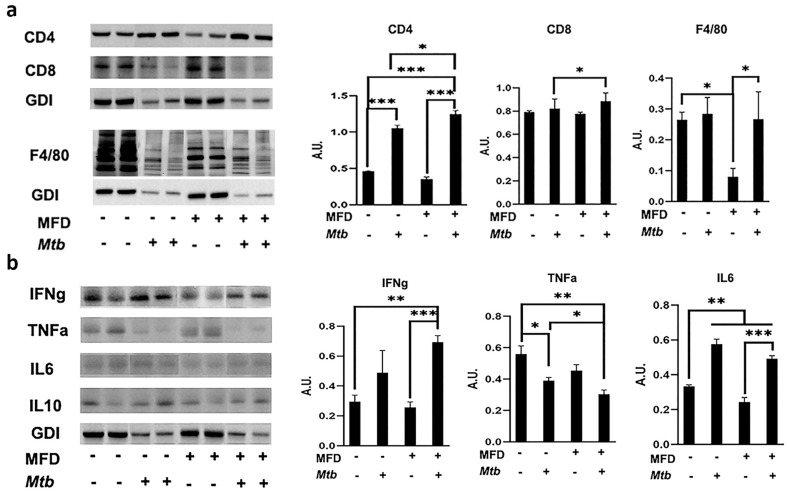
MFD alters the level of infiltrated immune cells and inflammatory markers in the lungs during chronic Mtb infection. Immunoblot analysis of (**a**) immune cells (CD4^+^, CD8^+^, and F4/80^+^) and (**b**) inflammatory markers (IFNγ, TNFα, and IL-6) in the lung lysates of RD-fed and MFD-fed uninfected and infected C57BL/6 mice during chronic (90 DPI) infection. The error bars represent standard error of the mean. * *p* < 0.05, ** *p* < 0.01, and *** *p* < 0.001 between indicated groups.

However, during the chronic stage, the levels of CD4^+^ cells increased in both RD- and MFD-fed mice compared to their respective diet-fed uninfected mice as demonstrated by Western blotting analysis (Figure 4a). The levels of CD8^+^ cells significantly increased in MFD-fed infected mice compared to RD-fed infected group during chronic infection (Figure 4a). The levels of F4/80^+^ cells were not altered between RD-fed infected and MFD-fed infected mice during chronic infection (Figure 4a). These data demonstrated that in RD-fed infected mice during acute infection, only F4/80^+^ cells significantly infiltrated the lungs, whereas during chronic infection, only CD4^+^ cells significantly infiltrated the lungs compared to uninfected mice. In contrast, in MFD-fed infected mice, both F4/80^+^ and CD4^+^ cells significantly infiltrated the lungs during both acute and chronic stages of infection compared to uninfected mice. Interestingly, in infected MFD-fed mice, the levels of CD8^+^ cells were significantly higher compared to infected RD-fed mice during both acute and chronic infections (Figure 3, Figure 4, and Supplementary Figure S4), suggesting that diets can regulate localized CD8^+^ cell levels.

We further analyzed the active status of infiltrated immune cells during chronic infection by measuring the levels of pro-inflammatory markers such as IFNγ, TNFα, and IL-6 in the lungs by Western blotting. During chronic infection, both IFNγ and IL-6 significantly increased in MFD-fed mice compared to uninfected groups. However, only IL-6 significantly increased in RD-fed infected mice compared to the uninfected group during the chronic stage (Figure 4b). However, the levels of TNFα significantly decreased in infected mice during chronic infection compared to uninfected groups in both RD- and MFD-fed mice (Figure 4b). These data demonstrated that MFD enhances the pro-inflammatory environment in the lungs during both acute and chronic stages of Mtb infection.

### 3.4. Mtb Infection Increases Adipogenesis and Alters Lipolysis in the Lungs Differently during Acute and Chronic Infection

**Figure 5:**
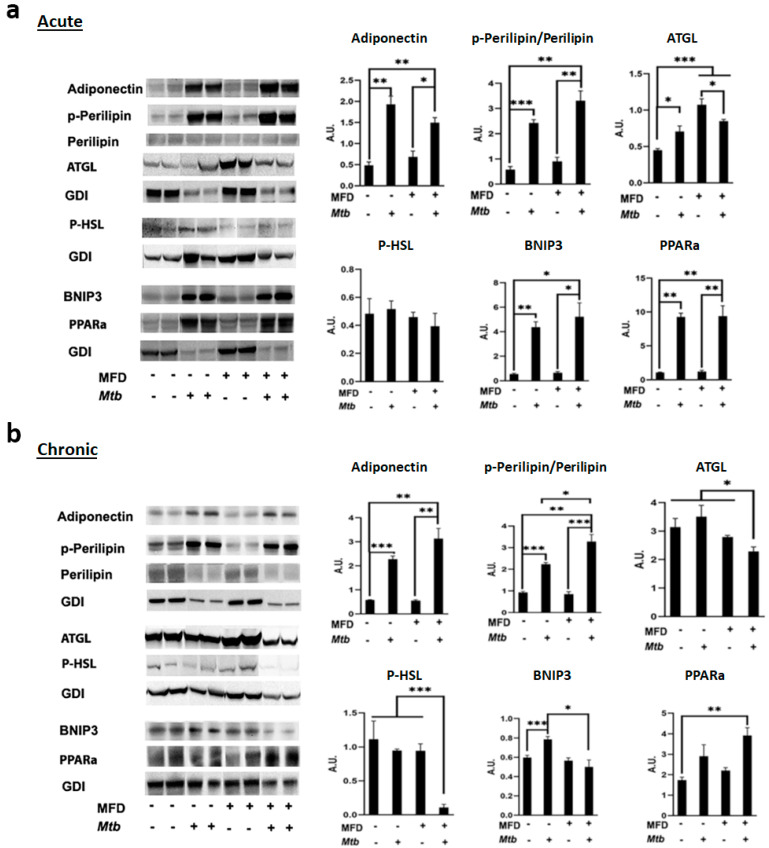
MFD increases lipid oxidation in the lungs during (**a**) acute and (**b**) chronic Mtb infection and protects from necrosis during chronic infection. Immunoblot analysis of adipogenesis marker (adiponectin), lipid oxidation marker (p-Perilipin, ATGL, p-HSL, and PPARα), and necrosis marker (BNIP3) in the lung lysates of RD-fed and MFD-fed uninfected and infected C57BL/6 mice during acute infection (30 DPI) and chronic (3 months) post-infection. The error bars represent standard error of the mean. * *p* < 0.05, ** *p* < 0.01, and *** *p* < 0.001 between indicated groups.

To analyze the effects of MFD on adipogenesis, lipolysis, and lipid oxidation in the lungs, we measured the protein levels of adiponectin (a marker of adipogenesis), p-Perilipin, ATGL, p-HSL (markers of lipolysis), and PPARα (marker of lipid oxidation) by Western blotting. The levels of adiponectin and p-Perilipin significantly increased in the lungs of both RD- and MFD-fed infected mice during both acute and chronic infections compared to uninfected mice (Figure 5). The levels of p-Perilipin significantly increased in MFD-fed infected mice compared to RD-fed infected mice during chronic infection, suggesting that lipid droplets are greatly degraded in MFD-fed infected mice (Figure 5b). The levels of ATGL significantly increased in the lungs of RD-fed infected mice but significantly decreased in the lungs of MFD-fed infected mice compared to their respective diet-fed uninfected mice during acute infection (Figure 5a). The levels of ATGL were not altered in RD-fed infected mice and significantly decreased in MFD-fed infected mice compared to their respective diet-fed uninfected mice during chronic infection. Interestingly, the levels of both ATGL and p-HSL significantly decreased in MFD-fed infected mice compared to RD-fed infected mice during chronic infection (Figure 5b). The levels of PPARα significantly increased in both RD-fed infected and MFD-fed infected mice during acute infection and only in MFD-fed infected mice during chronic infection compared to uninfected RD-fed mice (Figure 5). These data suggest that decreased levels of lipases during chronic infection might cause increased accumulation of lipid droplets in the lungs, which is supported by the histological data (Supplementary Figure S2). In addition, our data demonstrated that significantly increased lipid degradation and oxidation (detected by p-Perilipin and PPARα, respectively) in the lungs of infected MFD-fed mice compared to infected RD-fed mice likely reduces the accumulation of lipid droplets in the lungs of infected MFD-fed mice, as also supported by histological images (Supplementary Figure S2). It is known that the accumulation of intracellular lipids causes cell necrosis [15]. Thus, we examined the levels of necrosis marker BNIP3 in the lungs. Our Western blotting analysis demonstrated that the levels of BNIP3 increased in both RD- and MFD- fed infected mice compared to their respective diet-fed uninfected groups during acute infection and that BNIP3 significantly decreased in MFD-fed infected mice compared to RD-fed infected mice during chronic infection (Figure 5b). These data suggest that the signaling pathways leading to necrosis may be different between acute and chronic stages of infection. Acute inflammation may induce necrosis during acute infection, whereas changes in the lung lipid metabolism due to an increased accumulation of lipid droplets could be a cause for necrosis in chronic infection. It is possible that reduced levels of lipid accumulation in the lungs of infected MFD-fed mice might protect mice from severe lung necrosis during chronic Mtb infection.

### 3.5. MFD Alters Immune Signaling and Cell Death Pathways in Adipose Tissue during Acute and Chronic Mtb Infection

**Figure 6:**
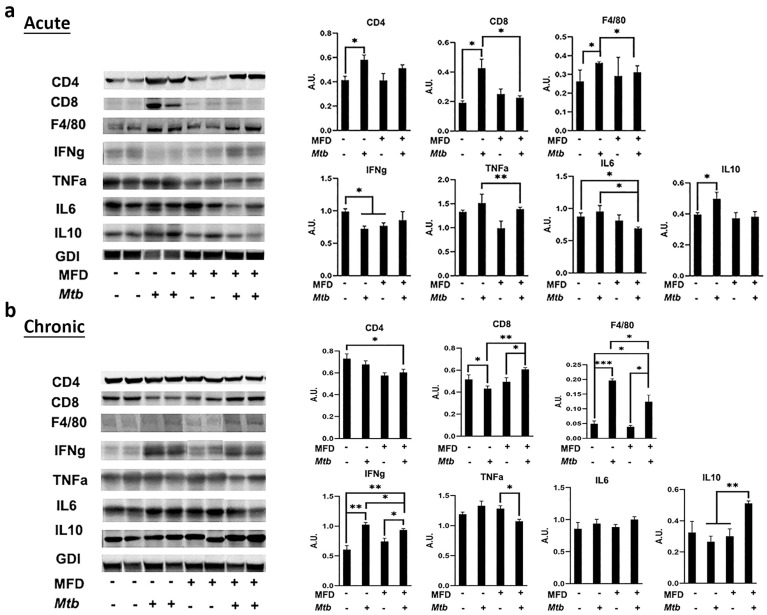
MFD alters immune signaling in adipose tissue during (**a**) acute and (**b**) chronic Mtb infection. Immunoblot analysis of immune cells (CD4, CD8, and F4/80) and inflammatory markers (IFNγ, TNFα, IL-6, and IL-10) in the WAT lysates of RD-fed and MFD-fed uninfected and infected C57BL/6 mice during acute infection (30 DPI) and chronic (3 months) post-infection is shown. The error bars represent standard error of the mean. * *p* < 0.05, ** *p* < 0.01, and *** *p* < 0.001 between indicated groups.

Previously, we demonstrated that Mtb infection alters adipose tissue physiology and that, conversely, a loss of body fat (adipocytes) affects pulmonary TB pathology [15]. To assess the effect of MFD on the infiltration of immune cells and inflammatory signaling in adipose tissue, we used Western blotting to analyze the levels of CD4^+^ cells, CD8^+^ cells, and F4/80^+^ cells (macrophages) in visceral fat isolated at 30 and 90 DPI (Figure 6). The levels of CD4 and CD8 significantly increased in infected RD-fed mice but not in infected MFD-fed mice compared to uninfected diet-matched mice during acute infection (Figure 6a). However, during the chronic stage of infection, the levels of CD4 remained unaltered and significantly decreased in infected RD-fed and infected MFD-fed mice, respectively, compared to RD-fed uninfected mice (Figure 6). Interestingly, the levels of CD8 significantly decreased in infected RD-fed mice and significantly increased in infected MFD-fed mice compared to their respective diet-matched uninfected mice during chronic infection. The levels of F4/80 significantly increased in infected RD-fed mice as compared to uninfected RD-fed mice at 30 DPI (Figure 6a). However, the levels of F4/80 were unchanged between infected and uninfected MFD-fed mice at 30 DPI. During the chronic stage, the levels of F4/80 significantly increased in both infected RD-fed and infected MFD-fed mice, as compared to diet-matched uninfected mice. Additionally, the levels of F4/80 in infected RD-fed mice were significantly higher compared to infected MFD-fed mice during chronic infection. The levels of pro-inflammatory cytokines, such as IFNγ, TNFα and IL-6, either were not significantly altered or were significantly decreased in both infected RD-fed mice and infected MFD-fed mice compared to their respective diet-matched groups in acutely infected mice (Figure 6a). However, IFNγ significantly increased in infected mice (both RD- and MFD-fed) compared to diet-matched groups during chronic infection (Figure 6b). Interestingly, the levels of anti-inflammatory cytokine IL-10 significantly increased in infected RD-fed mice during acute infection and significantly increased in infected MFD-fed mice during chronic infection. These data suggest that infection induces different immunological and inflammatory states in adipose tissue during acute and chronic Mtb infection, and that IFNγ signaling may differ between infected RD- and MFD-fed mice depending on the levels of IL-10.

**Figure 7:**
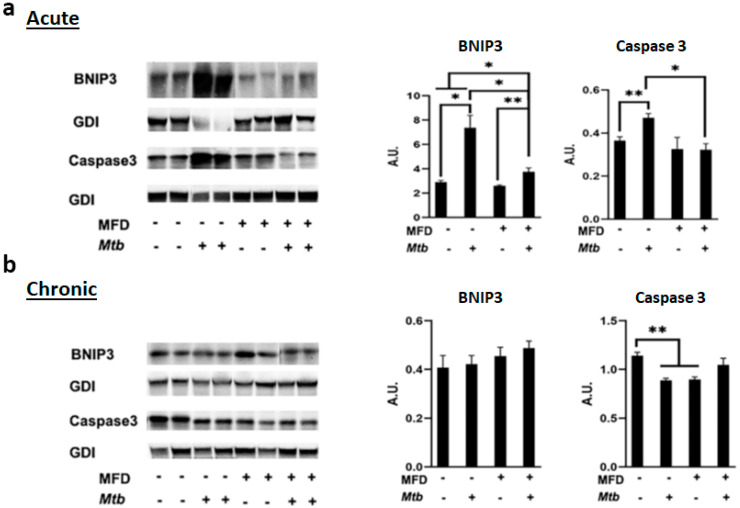
MFD alters cell death pathway in adipose tissue during (**a**) acute and (**b**) chronic Mtb infection. Immunoblot analysis of cell death markers, BNIP3 (marker of necrosis) and cleaved caspase (marker of apoptosis), in the WAT lysates of RD-fed and MFD-fed uninfected and infected C57BL/6 mice during acute infection (30 DPI) and chronic (3 months) post-infection. The error bars represent standard error of the mean. * *p* < 0.05 and ** *p* < 0.01 between indicated groups.

Previously, we demonstrated that Mtb infection causes a loss of adipose tissue due to cell death [4]. We analyzed whether death signaling differs between RD- and MFD-fed infected mice by measuring the levels of BNIP3 (a marker of necrosis) and cleaved caspase (a marker of apoptosis) by Western blotting. We observed that the levels of BNIP3 and cleaved caspase significantly increased in infected RD-fed mice and that only BNIP3 increased in infected MFD-fed mice compared to diet-matched uninfected mice during acute infection. The levels of BNIP3 and cleaved caspase significantly decreased in infected MFD-fed mice as compared to infected RD-fed mice during acute infection (Figure 7a). The levels of BNIP3 and cleaved caspase either were not altered or were significantly reduced in infected RD-fed and infected MFD-fed mice compared to diet-matched uninfected mice during chronic infection (Figure 7b). These data demonstrated that the loss of fat cells was greater in infected RD-fed mice compared to infected MFD-fed mice during acute infection, suggesting that MFD may protect mice from severe loss of body fat during acute infection.

### 3.6. Diets Differently Alter Serum Lipid Levels during Acute and Chronic Mtb Infection

**Figure 8:**
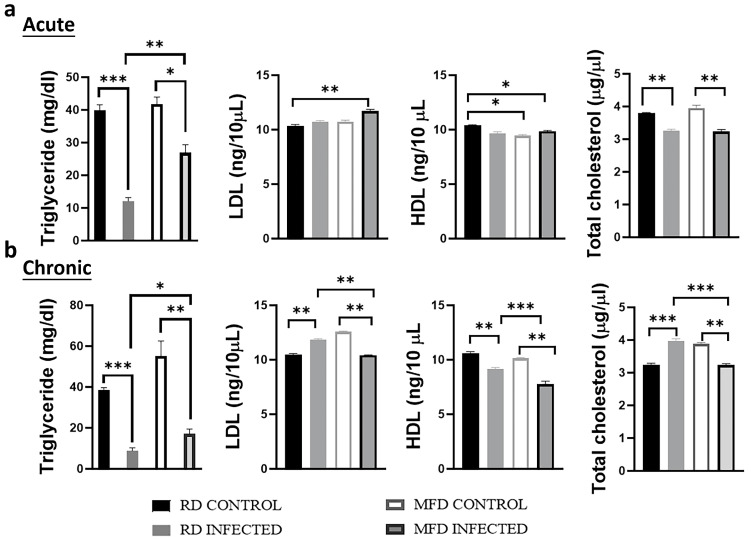
Altered serum lipid profile showing circulatory triglycerides, LDL, HDL, and total cholesterol of RD- and MFD-fed male C57BL/6 mice during (**a**) acute and (**b**) chronic Mtb infection. The error bars represent standard error of the mean. * *p* < 0.05, ** *p* < 0.01, and *** *p* < 0.001 between indicated groups.

As shown above, Mtb infection altered adipose tissue physiology differently in mice during acute and chronic infections and between mice fed on RD and MFD. As the loss of adipocytes due to necrosis and apoptosis may alter the circulatory lipid profile, we analyzed the serum levels of triglycerides, HDL, LDL, and total cholesterol as described in the Materials and Methods section (Figure 8). We found that Mtb infection significantly reduced the levels of triglycerides compared to uninfected groups during both acute and chronic stages of infection (Figure 8a,b). However, between the infected groups, the levels of triglycerides were significantly higher in MFD-fed infected mice compared to RD-fed infected mice (Figure 8a,b). During acute infection, the levels of LDL were not significantly altered in RD-fed infected mice but were significantly increased in MFD-fed infected mice compared to their respective diet-fed uninfected mice (Figure 8a). During the chronic stage, the levels of LDL significantly increased in RD-fed infected mice and significantly decreased in MFD-fed infected mice compared to their respective diet-fed uninfected mice (Figure 8b). Furthermore, MFD significantly decreased the levels of HDL in acute uninfected mice compared to RD-fed uninfected mice (Figure 8a). During chronic infection, the levels of HDL significantly decreased in both RD-fed and MFD-fed infected mice compared to their respective diet-fed uninfected mice (Figure 8b). The levels of HDL in MFD-fed infected mice were significantly reduced compared to RD-fed infected mice during chronic infection (Figure 8b). The levels of total cholesterol also significantly decreased in RD-fed infected and MFD-fed infected mice compared to the respective diet-fed uninfected mice during acute infection (Figure 8a). The levels of total cholesterol increased in RD-fed infected mice compared to RD-fed uninfected mice, whereas its levels significantly decreased in MFD-fed infected mice compared to RD-fed infected mice during the chronic stage (Figure 8b). The levels of total cholesterol in MFD-fed infected mice significantly decreased compared to RD-fed infected mice during the chronic stage (Figure 8b). Altogether, these data demonstrated that Mtb infection causes a reduction in serum levels of total cholesterol and triglycerides during acute infection irrespective of the diet fed. However, RD increases and MFD decreases serum cholesterol levels during the chronic stage compared to their respective diet-fed control mice.

### 3.7. Diets Differently Regulate Circulatory Cytokine Levels during Acute and Chronic Mtb Infection

**Figure 9:**
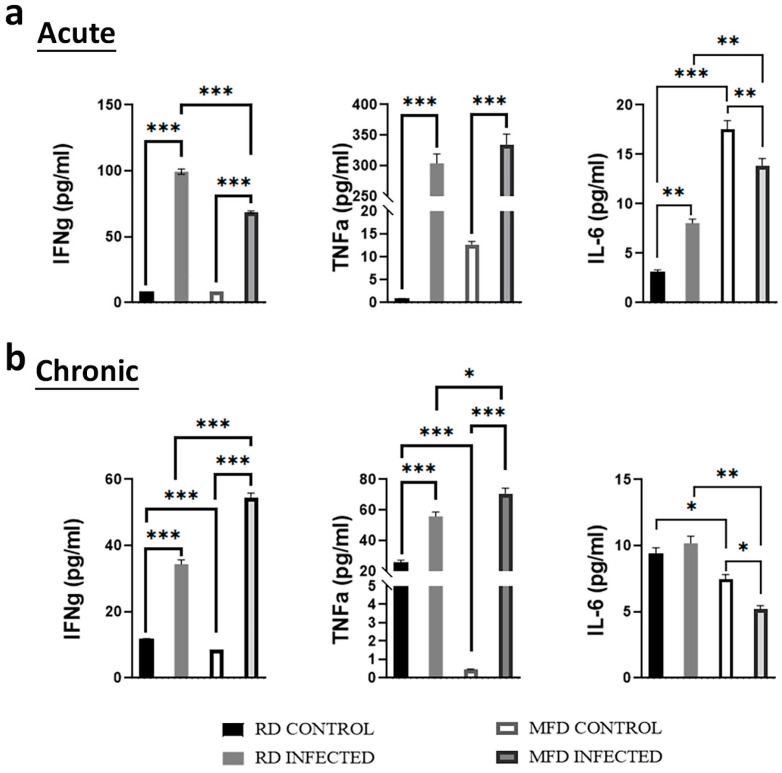
Analyses of circulatory cytokines (IFNγ, TNFα, and IL-6) in serum of RD- and MFD-fed control and infected C57BL/6 mice during (**a**) acute (30 DPI) and (**b**) chronic (90 DPI) infection by ELISA. The error bars represent standard error of the mean. * *p* < 0.05, ** *p* < 0.01, and *** *p* < 0.001 between indicated groups.

The data shown above (Figure 4, Figure 6 and Figure S4c) demonstrated that Mtb infection differently alters the levels of pro-inflammatory cytokines in the lungs and WAT during acute and chronic infection. For example, the levels of TNFα decreased in the lungs and increased in WAT of acute infected mice compared to uninfected mice. These infected tissues release cytokines, thus potentially altering systemic cytokine levels. To examine the effect of diet on circulatory cytokines during acute and chronic infection, we analyzed the levels of pro-inflammatory cytokines such as IFN-γ, TNF-α, and IL-6 in the serum of acute and chronic infected mice by ELISA (Figure 9). The serum levels of IFN-γ and TNF-α significantly increased in the infected mice fed on RD and MFD both during acute and chronic infections compared to the respective diet-fed uninfected mice. The levels of IFN-γ were significantly increased in RD-fed infected mice compared to MFD-fed infected mice during acute infection (Figure 9a); however, the levels of both IFN-γ and TNF-α significantly increased in MFD infected mice compared to RD infected mice during chronic infection (Figure 9b). Mtb infection significantly increased serum IL-6 levels in RD-fed infected mice compared to RD-fed uninfected mice during acute infection (Figure 9a). Although the serum levels of IL-6 significantly decreased in MFD-fed infected mice compared to MFD-fed uninfected mice, the levels of IL-6 were still significantly greater in MFD-fed infected mice compared to RD-fed infected mice during acute infection (Figure 9a). During chronic infection, the levels of IL-6 significantly decreased in MFD-fed infected mice compared to MFD-fed uninfected mice (Figure 9b). Interestingly, among the uninfected groups fed on different diets, we found that feeding a MFD for 8 weeks increased serum IL-6 levels compared to RD (acute time point) and that a prolonged feeding of MFD for approximately 17 weeks (chronic time point) decreased serum Il-6 levels compared to RD. These data demonstrated that MFD increases serum pro-inflammatory cytokines IFN-γ and TNF-α during chronic stages of Mtb infection.

### 3.8. Adipocytes and Adipocyte-Derived Lipid Droplets Alter Macrophage Activation during Mtb Infection

**Figure 10:**
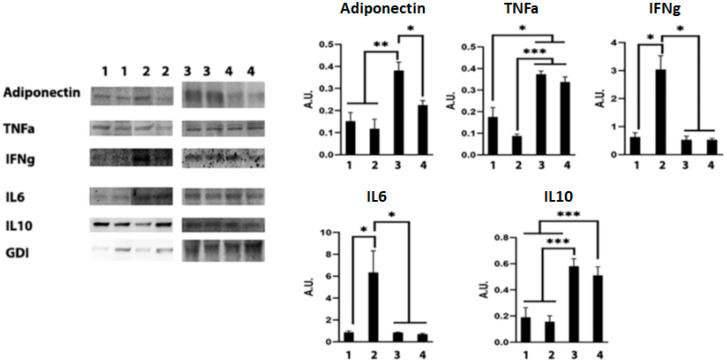
Adipocytes and adipocyte-derived lipid droplets alter macrophage activation during Mtb infection. Immunoblot analysis of adipogenesis marker (adiponectin) and immune markers (IL-6, IL-10, TNFα, and IFNγ) in the lysates of macrophages. Sample #1—uninfected RAW cells; Sample #2—infected RAW cells; Sample #3—infected RAW cells in contact with adipocytes; Sample #4—infected RAW cells in contact with adipocytes treated with recombinant TNF. The error bars represent standard error of the mean. * *p* < 0.05, ** *p* < 0.01, and *** *p* < 0.001 between indicated groups.

Previously, we demonstrated that an acute loss of fat cells increases the levels of lipid droplets and adipogenesis in the lungs during acute Mtb infection [4]. In this study, our data indicated that acute infection causes a significant loss of fat cells and a concomitant increase in the levels of adiponectin in the lungs (Figure 5). Increased adiponectin levels induce adipogenic signaling and formation of lipid droplets, which may result in lipotoxicity. We observed significantly increased lipase (ATGL) during acute infection (Figure 5). However, either reduced or unaltered levels of lipases in the lungs during chronic infection cause lipid accumulation and the formation of foamy macrophages in the lungs. We also observed significantly reduced levels of TNFα in the lungs during chronic infection, which correlated to the levels of foamy macrophages. These data suggested that increased adipogenic signaling and lipid droplets may regulate the activation of macrophages. Therefore, we hypothesized that adipocytes and lipid droplets may alter the activation of immune cells. To test our hypothesis, we performed in vitro experiments (detailed in the Materials and Methods section) using Mtb-infected RAW macrophages exposed to adipocytes via trans-well for 24 h (Supplementary Figure S1b). We used infected RAW cells cultured in plates (in the absence of contact with adipocytes) as the positive control (group 2, Supplementary Figure S1b). Uninfected RAW cells cultured in plates (in the absence of adipocytes) served as the experimental control (group 1, Supplementary Figure S1b). To examine the effects of adipocyte-released lipid droplets on macrophages, we induced apoptosis of adipocytes in trans-well by treating adipocytes with exogenous TNF (group 4, Supplementary Figure S1b) [16]. We analyzed the levels of adiponectin, pro-inflammatory cytokines (TNFα, IFNγ, and IL-6) and anti-inflammatory IL-10 by Western blotting analysis. Our data demonstrated that the levels of adiponectin significantly increased in trans-well cultured infected macrophages compared to plate cultured uninfected and infected macrophages (Figure 10). Pro-inflammatory cytokines, such as IL-6, IFNγ, and TNFα, were analyzed by Western blotting and found to be significantly increased in Mtb-infected macrophages (positive control) compared to uninfected macrophages during acute exposure (24 h) (Figure 10). However, the levels of IL-6, IFNγ, and TNFα significantly decreased in trans-well cultured infected macrophages compared to plate cultured infected macrophages (Figure 10). Interestingly, the levels of anti-inflammatory IL-10 significantly increased in trans-well cultured infected macrophages compared to plate cultured infected macrophages (Figure 10). These data show that macrophages in contact with adipocytes and lipid droplets likely express adiponectin, an anti-inflammatory and adipogenic protein that may regulate the levels of pro-inflammatory and anti-inflammatory status in infected macrophages.

### 3.9. Mtb-Infected Adipocytes Alter Immune Activation in Macrophages

Earlier, we demonstrated that Mtb infects adipocytes and adipose tissue and causes infiltration of macrophages in adipose tissue [4]. To examine whether Mtb-infected adipocytes regulate resident and infiltrated macrophages, we performed a trans-well experiment as described in the Materials and Methods section and analyzed the levels of adiponectin and inflammatory cytokines in RAW macrophages exposed to Mtb-infected adipocytes (group 6; Supplementary Figure S1b). Cultured macrophages exposed to uninfected adipocytes in the trans-well served as the control (group 5; Supplementary Figure S1b). Western blotting analysis demonstrated that the levels of pro-inflammatory cytokines such as IL-6, TNFα, and IFNγ significantly increased, and the levels of anti-inflammatory IL-10 were unaltered in macrophages exposed to infected adipocytes compared to macrophages exposed to uninfected adipocytes (Supplementary Figure S4). Interestingly, the levels of adipogenic protein adiponectin significantly decreased in macrophages exposed to infected adipocytes compared to macrophages exposed to uninfected adipocytes. These data show that infected adipocytes differently regulate macrophage activation compared to uninfected adipocytes (Supplementary Figure S4). Our data suggest that reduced adiponectin expression in macrophages likely induces M1 polarization and activation of pro-inflammatory signaling in macrophages. TNFα is known to promote pro-inflammatory signaling; however, the co-expression of high levels of IL-10 may induce M2 polarization and reduce pro-inflammatory effect of macrophages during acute infection, as indicated by the reduced levels of IL-6 and IFNγ (Figure 10).

## 4. Discussion

Several clinical studies have indicated that patients with active TB disease lose a significant amount of body weight, which is considered to be immunosuppressive and a major determinant of the severity and outcome of the disease [17]. Indeed, it has been shown that weight loss during TB treatment is a predictive marker of treatment failure [18]. Previously, using a transgenic murine Mtb infection model, we have demonstrated that loss of body fat is associated with increased pulmonary pathology and risk of reactivation of TB [10]. Our data indicated that loss of body weight in TB patients may be correlated to the loss of body fat, which plays an important role in TB pathogenesis and treatment outcome [19]. Therefore, in the present study, we examined whether increasing adiposity (fat mass) by feeding an MFD modulates pulmonary pathology during acute and chronic Mtb infections. We used Mtb HN878-infected C57BL/6 mice fed either an RD (containing 10% kcal fat) or an MFD (containing 30% kcal fat) and analyzed the effects of diet on pulmonary bacterial load, infiltration of immune cells, inflammation, and adipogenesis during acute and chronic infections. In addition, we examined the regulatory effects of uninfected or Mtb-infected adipocytes on the activation of uninfected or Mtb-infected macrophages by in vitro trans-well studies. Our data demonstrated that (i) MFD significantly reduces bacterial load in the lungs during acute infection compared to RD; (ii) RD and MFD differently alter the levels of infiltration of immune cells and activation of immune cells and lipolysis in the lungs during acute and chronic infections; (iii) MFD significantly increases the levels of infiltration of CD4^+^ and CD8^+^ cells during acute infection and only CD8^+^ cells in the lungs during chronic infection compared to RD-fed infected mice; (iv) MFD significantly increases the levels of pro-inflammatory IFNγ and IL-6 in the lungs during acute infection and significantly reduces the levels of IL-6 and TNFα during chronic infection compared to RD-fed infected mice; (v) MFD increases degradation and oxidation of lipid droplets and reduces necrotic cell death in the lungs during chronic infection compared to infected RD-fed mice; (vi) in infected mice, MFD decreases the levels of infiltration of CD8^+^ cells and macrophages in adipose tissue and reduces loss of fat cells during acute infection compared to RD; (vii) in infected mice, MFD significantly reduces inflammation in adipose tissue during chronic infections compared to RD; and (viii) adipocytes and lipid droplets induce M2 polarization in Mtb-infected macrophages via adiponectin signaling. These data suggest that during acute infection, MFD promotes a pro-inflammatory environment and infiltration of CD8^+^ cells in the lungs, likely causing a significant reduction in bacterial burden compared to RD-fed mice. Our data also suggest that MFD reduces inflammation and necrotic damage in the lungs during the chronic stage of infection compared to RD, and that these changes may involve adipocyte physiology and adipose tissue-derived anti-inflammatory IL-10. These data were supported by in vitro studies demonstrating that Mtb-infected adipocytes promote M1 polarization and activation of macrophages. It is particularly noteworthy that uninfected and infected adipocytes differently regulated macrophage polarization and activation.

The aerosol route of Mtb infection begins at the respiratory tract. Mycobacteria are ingested by resident alveolar macrophages in the alveolar space and the phagocytosed bacilli move from the alveolar space to the lung interstitium [20] and initiate innate and adaptive responses. Macrophages are effector cells of the innate immune system that phagocytose bacteria and secrete both pro-inflammatory and antimicrobial mediators. Mtb can also infect lung epithelial cells if the infiltrated macrophages are not able to completely phagocytose the bacterium and initiate infiltration of immune cells. Our data suggest that in the lungs, higher levels of innate response by macrophages are initiated in RD-fed mice, whereas an early higher level of adaptive response by CD4^+^ and CD8^+^ cells is initiated in MFD-fed mice during acute infection. The increased CD4 and CD8 levels and pro-inflammatory signaling in the lungs may help contain bacterial dissemination and reduce bacterial loads in MFD-fed mice during acute infection. It has been shown that the recruitment of CD4^+^ cells in the lungs is essential for the development of a protective granulomatous response to pulmonary TB [21]. Such an increase in CD4^+^ cells in the lungs may have caused the elevated formation of mature granulomas during the chronic stage of infection in both the RD-fed and MFD-fed infected mice (Figure 3). Increased CD4^+^ cells in the lungs of MFD-fed mice during acute and chronic stages of infection likely caused the elevated levels of IFNγ and initiated host survival mechanisms (Figure 4) [22]. Interestingly, the burdens of bacterial load in the lungs were not significantly different between infected RD-fed and infected MFD-fed mice during chronic stages of infection, as demonstrated by CFU count and AR-staining (Figure 2). Many factors, including genetic factors, may influence the host’s ability to constrain Mtb growth in the lungs, to avoid excessive immune-mediated lung tissue damage, or both [23]. It has been shown that disease severity and the rate of mortality may depend more upon the inflammatory response of the host rather than the direct toxicity of mycobacteria and their products [23]. Our data demonstrated that IL-6 and IFNγ-induced inflammatory response may dominate in the lungs of MFD-mice during chronic infection. Although IL-6 and IFNγ are considered to be pro-inflammatory, in the absence of TNFα (Figure 4), they may induce an anti-inflammatory response to protect host cells from cell death [24,25], as indicated by the reduced levels of the necrosis marker in the lungs compared to RD-fed infected mice (Figure 5).

Necrotic lesions in the lungs are hallmarks of pulmonary TB pathology, which constitute a severe health issue even in post-TB conditions. Necrosis contributes to the loss of lung function and is characterized by limited penetration of leukocytes and antibiotics, providing a secure niche for bacteria [26]. We observed increased levels of necrosis marker BNIP3 in the lungs of both infected RD-fed and infected MFD-fed mice during acute infection, which may be due to the increased TNFα levels in the lungs [4]. However, the levels of TNFα were reduced in the lungs during chronic infection in both RD-fed and MFD-fed mice, and our data indicated increased necrosis in infected RD mice. MFD significantly decreased necrosis (Figure 5) in the lungs compared to RD during chronic Mtb infection. This may be due to increased lipid hydrolysis and oxidation of accumulated lipid droplets in the lungs, as demonstrated by increased p-Perilipin and PPARα levels in MFD-fed chronic mice (Figure 5). The accumulation, hydrolysis, and oxidation of lipids may impact the activation of immune cells. Our data suggest that the mechanisms involved in necrosis signaling during acute and chronic infections may be different, with TNFα-induced necrosis present during acute infection and altered lipid metabolism and lipotoxicity-induced necrosis present during chronic infection. Thus, the host response to Mtb may be a major determinant of disease severity and outcome [23], with diet being able to alter the metabolic and immune responses in the lungs during acute and chronic infections.

Our data demonstrated that diets differently alter the levels of circulatory lipids during Mtb infection. Specifically, during acute infection, the serum levels of both the total cholesterol and triglycerides decreased compared to uninfected groups, whereas during the chronic stage, the levels of total cholesterol increased in RD-fed infected mice. It has been shown that lipids can alter pro-inflammatory cytokines [27,28,29]. Therefore, we analyzed the circulatory pro-inflammatory cytokines such as TNF-α, IFN-γ, and IL-6 during acute and chronic infections. Mtb infection (during both acute and chronic stages) in general increased the levels of TNF-α and IFN-γ in infected mice compared to uninfected mice irrespective of the diets fed, whereas the levels of TNF-α and IFN-γ significantly increased in MFD-fed mice during the chronic stage. However, the levels of IL-6 were reduced in infected MFD-fed mice compared to uninfected MFD-fed mice during both acute and chronic stages of infection. These data suggest that a prolonged MFD feeding may improve the circulatory levels of TNF-α and IFN-γ and not IL-6 during Mtb infection. It should be noted that the lipid profiles are species specific, and the proportion of blood LDL, HDL, and total cholesterol differs between humans and mice [30]. Thus, further correlative studies in patients with TB are warranted.

Previously, we showed that increased loss of body fat correlated to elevated pulmonary bacterial load and presence of foamy macrophages [10]. In this study, we demonstrated that MFD reduces fat loss during acute infection compared to RD. This may cause reduced lipid accumulation in the lungs, in turn reducing the formation of foamy macrophages and improving immune signaling. We showed significantly reduced bacterial burden in the lungs of MFD-fed mice compared to RD-fed mice during acute infection. Increased loss of adipocytes and accumulation of lipid droplets in the lungs alter the activation of immune cells. Our in vitro studies demonstrated that the macrophages in contact with adipocytes show M2 polarization and express lower levels of pro-inflammatory cytokines when infected with Mtb compared to infected macrophages (without adipocyte contact). Our data indicate that lipid droplets can induce adiponectin expression in macrophages, which likely altered the activation status of the macrophages.

## 5. Conclusions

In conclusion, our data suggest that slight/moderate adiposity decreases the risk of active TB disease by increasing the activation of immune cells in the lungs, reducing the bacterial load, and containing bacterial dissemination. In addition, slight adiposity could protect subjects with chronic latent TB infections from activation/reactivation of TB disease by regulating immune and metabolic functions in the lungs. Further mechanistic studies are warranted to understand the effects of various fat-rich diets on the pathophysiology of adipose tissue and the risk of TB activation in the TB–DM scenario.

## Data Availability

Not applicable.

## Supplementary Materials

The following supporting information can be downloaded at: https://www.mdpi.com/article/10.3390/life13010228/s1, Figure S1: (a) Flowchart of experimental design; (b) overlay of in vitro experiment; (c) percent weight change in acute infected mice. The error bars represent standard error of the mean. * *p* < 0.05 and ** *p* < *0*.01 between indicated groups. Figure S2: Histological analysis of the lungs in H&E sections of lungs in chronic Mtb infection. Red arrow indicates necrosis, green arrow indicates lipid droplets, and black arrows indicate foamy macrophages. The images are 40X magnification. Figure S3: Immunoblot analysis of immune cells (CD4^+^, CD8^+^, and F4/80^+^) in the lung lysates of RD-fed and MFD-fed, male and female, uninfected and infected C57BL/6 mice during acute infection (30 DPI) and chronic (90 DPI) infection. The error bars represent standard error of the mean. * *p* < 0.05 between indicated groups. Figure S4: (a) Representative IHC images of the lung sections of mice during chronic Mtb infection (90 DPI) stained by CD4^+^, CD8^+^, F4/80^+^, and IFNγ antibodies. (RD_con—uninfected mice fed an RD; MFD_con—uninfected mice fed an MFD; RD_Inf—infected mice fed an RD; and MFD_Inf—infected mice fed an MFD). Magnification—4×; Scale bar—100 µ. (b) Bar graphs showing histological grading of antibody-stained (CD4^+^, CD8^+^, F4/80^+^, and IFNγ+) lung sections. Each class was graded on a 7-point scale ranging from 0 to 6 as discussed in the Materials and Methods section. The error bars represent the standard error of the mean. * *p* < 0.05 and ** *p* < 0.01 between indicated groups. (c) Immunoblot analysis of immune cells (CD4^+^, CD8^+^, and F4/80^+^) and inflammatory cytokines (IFNγ, TNFα, and IL-6) in the lung lysates of RD-fed and MFD-fed uninfected and infected C57BL/6 mice during acute (30 DPI) infection. The error bars represent standard error of the mean. * *p* < 0.05 and ** *p* < 0.01 between indicated groups. Figure S5: Mtb-infected adipocytes alter immune activation in macrophages. Immunoblot analysis of adipogenic marker (adiponectin) and immune markers (IL-6, IL-10, TNFα, and IFNγ) in the lysates of macrophages. Sample #5—uninfected RAW cells with uninfected adipocytes (in trans-well); Sample #6—uninfected RAW cells with infected adipocytes (in trans-well). The error bars represent standard error of the mean. ** *p* < 0.01 and *** *p* < 0.001 between indicated groups.
