# Supplementary figures and images for: Diets Differently Regulate Pulmonary Pathogenesis and Immune Signaling in Mice during Acute and Chronic Mycobacterium tuberculosis Infection
Source: Life (Basel). 2023 Jan 13;13(1):228. doi: 10.3390/life13010228 (PMC9861969; doi:10.3390/life13010228)

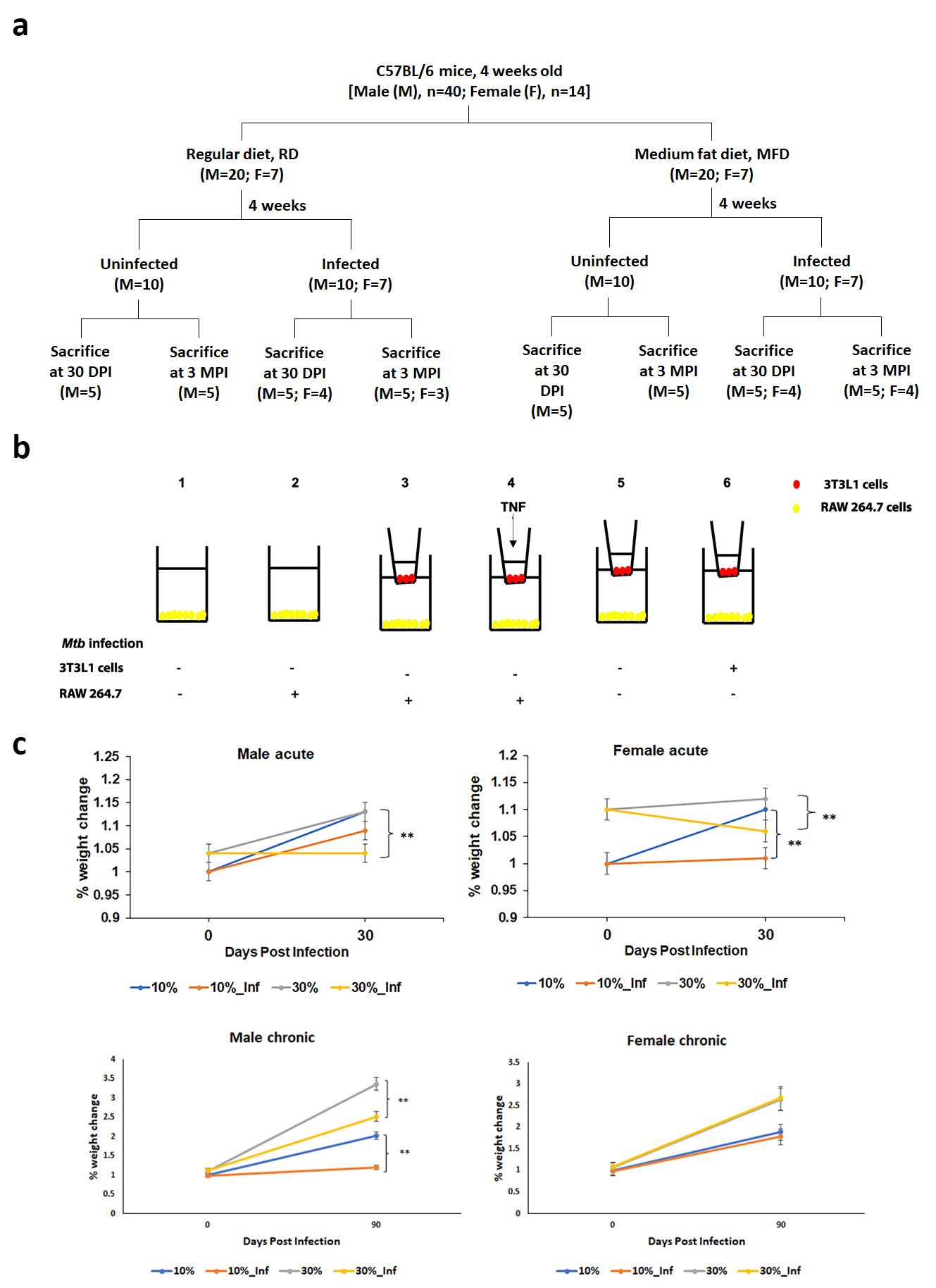

Supplement: Supplementary file 1 [file life-13-00228-s001.zip › Supplementary figures/Fig S1.tif]

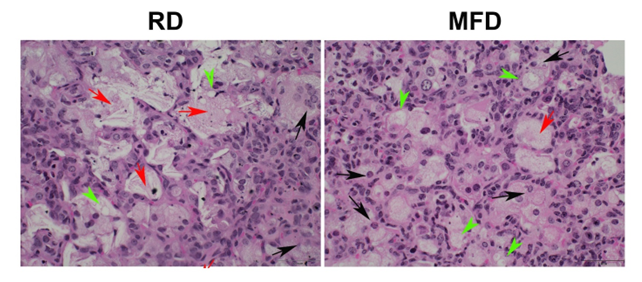

Supplement: Supplementary file 1 [file life-13-00228-s001.zip › Supplementary figures/Fig S2.tif]

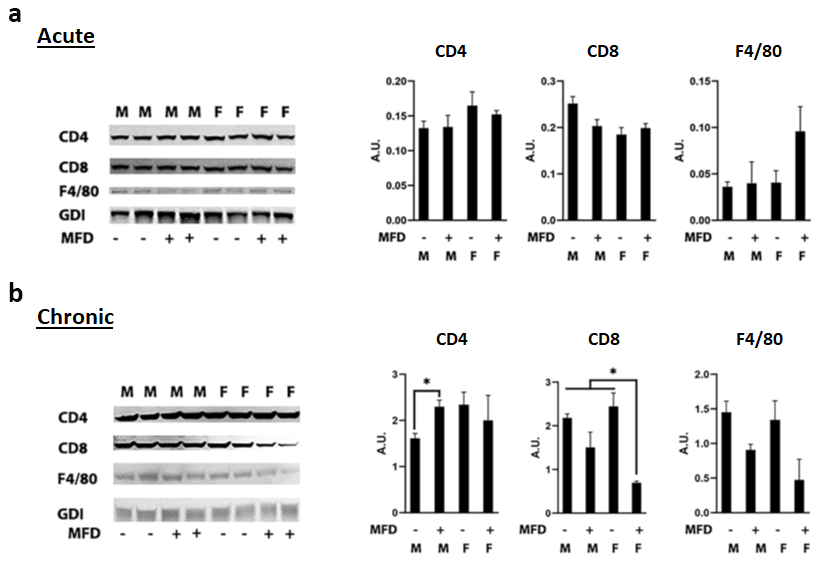

Supplement: Supplementary file 1 [file life-13-00228-s001.zip › Supplementary figures/Fig S3.tif]

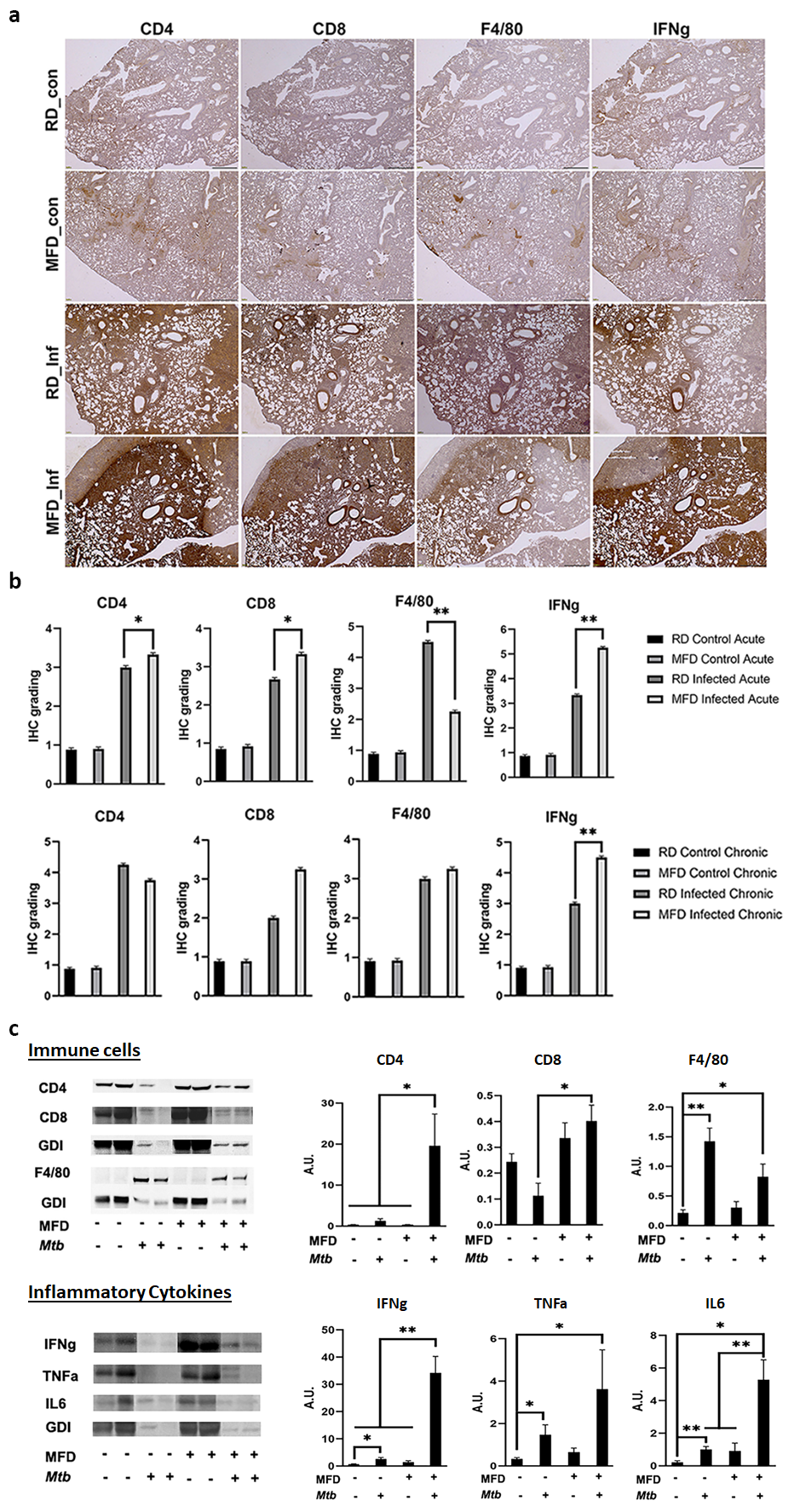

Supplement: Supplementary file 1 [file life-13-00228-s001.zip › Supplementary figures/Fig S4.tif]

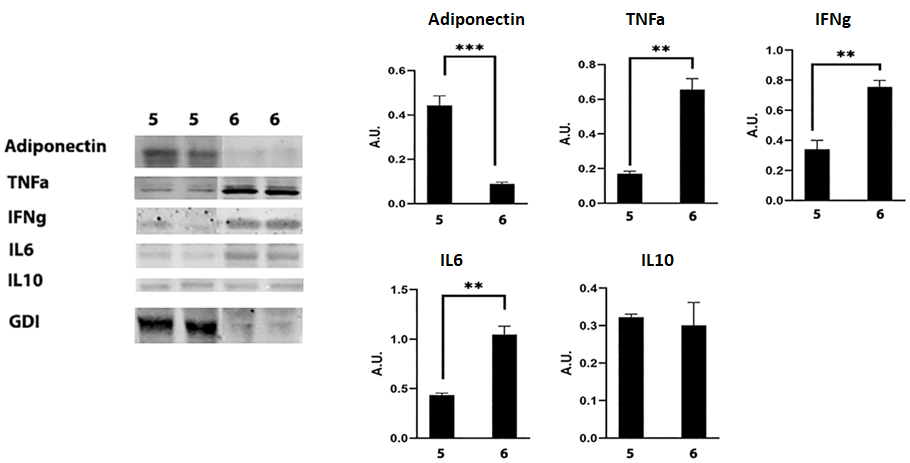

Supplement: Supplementary file 1 [file life-13-00228-s001.zip › Supplementary figures/Fig S5.tif]
